# Applying the unified pH scale: absolute acidities in the gas phase and anchor points for eleven representative liquid media

**DOI:** 10.1186/1758-2946-4-S1-P8

**Published:** 2012-05-01

**Authors:** Sascha K Goll, Daniel Himmel, Ivo Leito, Ingo Krossing

**Affiliations:** 1 Institute for Inorganic and Analytical Chemistry, Freiburger Materialforschungszentrum FMF and Freiburg Institute for Advanced Studies (FRIAS), Albert-Ludwigs-Universität Freiburg, Freiburg, 79104, Germany; 2 Institute of Chemistry, University of Tartu, Tartu, 50411, Estonia

## 

The investigations on our recently introduced unified acidity scale [1] based on the absolute chemical potential of the proton pointed out the inadequateness of the established GA scale. Earlier it was inter alia found that, when trying to correlate p*K*_a_ with GA values, in several cases the correlation was broken without any sufficient explanation [2,3]. However, the GA does not take into account the pressure dependent speciation in the gas phase. In this contribution we systematically extend the theory of acidity in the gas phase from standard GAs and GBs to the real existing bulk phases [4].

Furthermore we present the *rCCC* (relaxed COSMO cluster-continuum) model [5], a quantum chemical solvation model for the calculation of *Gibbs* solvation energies of the proton with good accuracy. The *rCCC* values can be used to anchor individual pH scales in different solvents to our universal scale.
